# Dogs and the Community

**DOI:** 10.4269/ajtmh.24-0607

**Published:** 2025-02-04

**Authors:** Angel Sebastian Rodriguez-Pazmiño

**Affiliations:** One Health Research Group, Universidad de Las Américas, Quito, Ecuador

Since I was little, I have always been close to pets. Whenever I see a starving dog, I try to feed it. If a dog is injured, I take it to a veterinarian. These concerns for stray dogs became even more evident when I started college in 2014 at Yachay Tech University, Urcuquí, Ecuador, where I was part of the university’s first generation of students.

During my second semester, after finishing a class, I said to my friend Denisse Andrade, “Hey, I think I saw a dog go through a hole in the wall into some wooden houses before class. Would you come with me to check it out?” I had a strong feeling that we would find something interesting.

We had to ask some guards to help us open the gate when we arrived at the site. Once inside, we discovered a yellow dog lying on the wooden floor nursing five tiny puppies that could not have been more than a week old. Denisse gasped and covered her mouth in surprise. I turned to her and said, “We have to name her. You’re going to be called … Curie” (after the scientist who won two Nobel Prizes).

It was not the first time that someone had encountered dogs in that situation on campus, but there was a growing sense that it was happening more frequently. The semester was almost over, yet Denisse and I were determined to find ways to care for them. During the vacation period, the guards helped us by feeding the dogs with the food that we left behind. We also spoke with a woman who owned a nearby restaurant and asked if she could occasionally leave some of her leftover food for them.

One Friday night, over beers at a karaoke bar, we shared our experience with Curie with some friends. María José De la Torre, a student from the second generation, said, “I can’t sleep well at night. When one dog starts howling, five others join in, like they’re performing at a concert. But I can’t stay mad at them. In the mornings, when I go to class, there’s always one that walks with me and waits outside until I’m done. I’ve grown fond of them.” By that time, a population of about 40 dogs had already settled across the campus, with many choosing to spend the night near the student residences.

Lenin Rueda, a friend from the first generation, remarked, “Some professors really dislike them. They can’t stand having the dogs around during class—either because of their smell or simply because they don’t want to see them.” In truth, most people enjoyed the dogs’ company. However, we had to admit that their presence sometimes caused discomfort. During mealtimes at the university restaurants, one or two dogs would often linger by the tables, waiting for scraps. Occasionally, garbage bags were torn open as the dogs searched for food. There were even a few instances of people being bitten.

“Well, let’s start a club of students to do something for the dogs. Enough talk,” I told my friends, inspired by an animal welfare group that I had supported back in high school. María José enthusiastically replied, “Let’s do it!” The others seemed on board as well. It took us several months of planning, but on May 30, 2016, we inaugurated the club. We named it the Club de Bienestar Animal de Yachay Tech—CBA for short.

We continued the activities that we had started in our first semester at the university but with greater consistency and organization. These included providing veterinary care for dogs in need, vaccinations, adoptions, and sterilization campaigns held in nearby cantons. Much of our volunteer time was also dedicated to fundraising efforts.

As the months passed, however, the results felt discouraging at times. Despite all of our efforts, the number of dogs on campus remained about the same, and their nighttime howling continued. Yet, there was one significant change; no dogs were giving birth on campus anymore. It was a small but meaningful victory that kept us motivated.

We were doing our best, and deep down, I think that we hoped for some recognition from the university authorities. Instead, we often faced criticism. We would hear comments in the hallways like “The CBA students are the ones bringing the dogs here” or “They’re giving the university a bad image.” Two professors even criticized our sterilization efforts, arguing that it was immoral to take away the dogs’ “right to procreate.” This remark ignored the reality in developing countries like Ecuador, where sterilization is essential to controlling the overwhelming number of abandoned pets.

At times, it felt like the challenges were piling up. Planning meetings were often attended by only one or two people—or occasionally, no one at all. The initial excitement surrounding the CBA had started to fade, and I found myself with plenty of reasons to give up.

After a biology class, Professor Javier Álvarez—someone I remember fondly—asked me, “Why do you look so down, Sebas?” I opened up to him about everything: the negative comments from some authorities, the lack of support, and how disheartening it all felt. He listened carefully and then said, “Nothing good comes easy. But let’s see if we can find a solution.”

The class had ended around 5 p.m., but we stayed in the classroom talking until nearly 8:30 p.m. Michelle Sánchez, a friend from the fifth generation, joined the conversation. Two dogs were with us, lounging on the chairs. They seemed to be listening too, occasionally glancing up as if they understood every word.

We wrote a series of key words on the blackboard to break down the root of the problem: “Yachay Tech, new ecological niche,” “late changes,” “complex system,” “lack of education,” “institutional irresponsibility,” and “poverty.” That brainstorming session helped us realize that Yachay Tech had inadvertently created a new ecological niche, one that was ideal for dogs.

The university, recently built, lacked any sort of perimeter enclosure. Its surroundings were primarily agricultural, with most households in the nearby communities having dogs. As is common in many parts of the country, these animals often live in poor conditions and roam freely in search of food. When they discover a place offering better resources and safety, they settle there—just as they had at Yachay Tech.

The reason that the dog population did not spiral out of control was twofold; our sterilization efforts curbed their reproduction, and the resident dogs naturally deterred new ones from entering their territory.

Much of the conversation with Professor Javier and Michelle in that classroom was spent reflecting on our understanding of the problem, but we knew that we needed to take action. That is when we decided to organize an Animal Welfare Fair through the CBA, which we held in December 2017.

The event featured a variety of activities: a cutest pet contest, a musical performance, the sale of vegetarian and vegan food, talks on responsible pet ownership, and of course, the centerpiece—a sterilization campaign. This idea helped reignite students’ enthusiasm for the club, and we ended up having a productive planning meeting with nearly 30 attendees. We were excited by the possibility that about a thousand people from Yachay and the surrounding communities might come to the fair.

When the day of the fair arrived, only about 200 people attended. It was a tough blow to our expectations. However, Irene Corrales, a close friend and one of the few administrative staff who supported the CBA unconditionally, quickly lifted our spirits with her charisma and natural ability to connect with people. With her and other club members, we made an effort to ensure that the attendees from the surrounding communities felt welcome and understood that we were there to support them in any way we could.

Some were unfamiliar with the concept of sterilization, whereas others could not afford the $20 fee. Thankfully, we were able to raise enough funds to offer many sterilizations for free. It was truly inspiring to witness the dedication of the local residents as they brought their pets—some walking from far-off areas, others arriving on motorcycles, and a few even transporting their animals in wheelbarrows typically used for agricultural or construction work.

That fair was the most significant event organized by the CBA during my time at the helm. Afterward, Michelle took over the club and continued the initiatives. Since then, three other leaders have guided the club at different times: Susset Navarro, Franshesca Vaca, and Jeremmy León. In a recent conversation with Jeremmy, the current leader, we reflected on how many of the challenges that I have described in this story are persisting. It seems like a never-ending task.

Nevertheless, the CBA’s enduring presence over the years keeps the hope alive that, little by little, we can make progress and continue having a positive impact on the Yachay Tech campus—and most importantly, on the communities of Urcuquí. It is essential to continue persevering.

